# Potential Mechanisms of COVID-19 Related Nervous System Damage and Effects on Female Fertility

**DOI:** 10.2174/0109298673333968241011092801

**Published:** 2024-10-24

**Authors:** Chen-yue Qian, Si-ning Hu, Hua-dong Liu, Jing-jin Liu

**Affiliations:** 1 The Second Clinical Medical College, Jinan University, Shenzhen, Guangdong, China;; 2 Key Laboratories of Education Ministry for Myocardial Ischemia, Department of Cardiology, Second Affiliated Hospital of Harbin Medical University, Harbin, Heilongjiang, China;; 3 Department of Cardiology, Shenzhen People's Hospital, The First Affiliated Hospital, The Second Clinical Medical College, Jinan University, Southern University of Science and Technology, Shenzhen, Guangdong, China;; 4 Shenzhen Cardiovascular Minimally Invasive Medical Engineering Technology Research and Development Center, Department of Cardiology, Shenzhen People's Hospital, The First Affiliated Hospital, The Second Clinical Medical College, Jinan University, Southern University of Science and Technology, Shenzhen, Guangdong, China;; 5 Shenzhen Key Laboratory of Stem Cell Research and Clinical Transformation, Shenzhen People’s Hospital, The First Affiliated Hospital, The Second Clinical Medical College, Jinan University, Southern University of Science and Technology, Shenzhen, Guangdong, China;; 6 Department of Geriatrics, Shenzhen People's Hospital, The First Affiliated Hospital, The Second Clinical Medical College, Jinan University, Southern University of Science and Technology, Shenzhen, Guangdong, China

**Keywords:** COVID-19, central nervous system, immune response, fertility, pharmacological treatment, ataxia

## Abstract

Signs and symptoms that persist or worsen beyond the “acute COVID-19” stage are referred to as long-COVID. These patients are more likely to suffer from multiple organ failure, readmission, and mortality. According to a recent theory, long-lasting COVID-19 symptoms may be caused by abnormal autonomic nervous system (ANS) activity, such as hypovolemia, brain stem involvement, and autoimmune reactions. Furthermore, COVID-19 can also cause impaired fertility in women, which may also be related to inflammation and immune responses. Currently, few treatments are available for long-COVID symptoms. This article reviews the major effects of COVID-19 on the nervous system and female fertility, as well as offers potential treatment approaches.

## 1 INTRODUCTION

The virus responsible for coronavirus disease 2019 (COVID-19), severe acute respiratory syndrome coronavirus 2 (SARS-CoV-2), has sparked a pandemic that has had hitherto unheard-of social, economic, and health effects worldwide. After 1 year, SARS-CoV-2 has infected >175 million people worldwide, and >3.8 million individuals have died from COVID-19 [1]. COVID-19 patients, along with presenting its characteristic systemic symptoms, may also have neurological symptoms, such as headache, vertigo, hypogeusia, hyposmia, myalgia, ataxia, and seizures [1, 2]. Indeed, in a 2021 cohort study, 82% (3069/3743) of COVID-19 patients reported neurological complaints [3], indicating that SARS-CoV-2 could infect and cause damage to the central nervous system (CNS). This pathological outcome is further supported by another retrospective case study of 214 hospitalized COVID-19 patients in Wuhan, China, in which >36.4% of patients had acute cerebrovascular disease, skeletal muscle damage, and disturbance of consciousness, as well as neurological symptoms, such as headache, nausea, dizziness, and hypogeusia [4]. Therefore, the impact of SARS-CoV-2 on the CNS, as well as the underlying mechanisms contributing to COVID-19 neurologic symptoms, has become a topic requiring additional research consideration [4]. In this review, we outlined those underlying mechanisms, namely, hypovolemia, brain stem involvement, and autoimmunity, and their effects on nervous system function. We also examined how SARS-CoV-2 infection affects female fertility, as well as pharmacological and non-pharmacological treatment approaches.

## 2 MECHANISMS CONTRIBUTING TO AUTONOMIC DYSFUNCTION ASSOCIATED WITH LONG-COVID

Interruptions, or increased sympathetic/parasympathetic nerve activity in the autonomic nervous system (ANS), serve as an indication of autonomic dysfunction, which presents with a range of clinical symptoms, such as fatigue, unstable blood pressure, postural hypotension, heart rate variability disorder (HRV), impotence, bladder dysfunction, and intestinal impairments. Furthermore, ANS disorders have been linked to sleep disturbances, as well as metabolic alterations impacting both endocrine and immune systems. This results in increased catecholamine secretion and subsequently, vasoconstriction, accelerated breathing, along with increases in blood pressure, triacylglycerols, plasma-free fatty acids, and platelet viscosity. All these changes, in turn, could lead to coronary heart disease, heart failure, hypertension, arrhythmia, plus other physiological and biochemical changes [5]. Autonomic dysfunction could manifest as sudden, gradual, chronic, or even reversible [6-8]. Hypovolemia, brain stem involvement, and autoimmunity have been tentatively identified as the likely underlying mechanisms behind SARS-CoV-2 causing this phenomenon [9-14] and are described below in detail.

### 2.1 Hypovolemia

Patients with long-COVID-19 and dys-autonomic symptoms frequently exhibit hypovolemia [13, 14], which may be due to the combination of fever, anorexia, nausea, night sweats, and extended bed rest possibly decreasing blood volume, and subsequently increasing cardiac sympathetic noradrenergic system (SNS) outflow [10, 15]. Subsequently, hypovolemia may cause hyperadrenergic postural orthostatic tachycardia syndrome (POTS) [16]. POTS individuals have been found to have lower plasma volumes, as well as renin and aldosterone levels, suggesting that deficiencies in the renin-angiotensin-aldosterone system (RAAS) may play a part in its etiology [17-19], which likely stems from partial renal sympathetic denervation [17].

Hypovolemia can also disrupt central autonomic networks and cause cerebral hypoperfusion, yielding reductions in cerebrospinal fluid (CSF) volumes and impaired cerebral blood flow, leading to structural and functional changes in the cortex and brainstem. Furthermore, systemic hypovolemia could contribute to increases in sympathetic outflow in response to central autonomic activation [20]. Post-COVID POTS, though, could be treated by ingesting large amounts of water and salt [21], as well as other non-pharmacological treatments aimed to increase venous return and intravascular volumes, as shown in preliminary studies [16].

### 2.2 Brain Stem Involvement

SARS-CoV-2 infection has been linked to brain stem dysfunction *via* direct viral invasion [22], neuroinflammation [23], and vascular activation [24]. As a coronavirus, SARS-CoV-2 is an encapsulated, single-stranded RNA virus, spherical in shape and possessing club-shaped surface spikes. Its genome encodes 4 primary structural proteins: nucleocapsid (N), envelope (E), membrane (M), and spike (S) [25], and the residual viral genome codes for a number of accessory proteins that disrupt the host's innate immune response [26]. The Spike-glycoprotein is the most important of the three protein components that make up the viral envelope. The site of entrance for SARS-CoV-2 into host cells is when the spike protein recognizes the host cells [26]. The S protein, comprising of S1 and S2 subunits [26], is involved in viral entry and binding to host cell surface receptors. More specifically, S1 possesses a receptor-binding domain that interacts with host angiotensin-converting enzyme 2 (ACE-2) receptor [27], while S2 promotes viral-cell membrane fusion. Due to its role in facilitating viral infection, the S protein has become the predominant target for vaccine development [28]. As for N, E and M proteins, E and N are important in viral assembly and budding, while M determines the shape of the viral envelope.

The spike protein is essential for identifying the ACE2 receptor on the host alveolar epithelial cells, and this is true of both SARS-CoVs and other SARS viruses in terms of pathogenesis [26]. Within the respiratory system, SARS-CoV-2 initially attaches to respiratory epithelial cells, multiplies, and descends to the alveolar epithelial cells. This initial attachment is *via* binding to the enzymatic domain of the ACE-2 receptor, and the inhibition of the ACE-2 peptidase activity, as well as fast replication by the virus, could trigger a robust immunological response [29]. ACE-2 is also present in various other cell types, such as neurons, intestinal epithelial, kidney, endothelial, monocytes/macro- phages, and neuroepithelial cells [30, 31]. Upon S protein attachment to the ACE-2 receptor, SARS-CoV-2 is endocytosed and transported into host endosomes, followed by cleavage of the S protein by transmembrane protease serine 2, cathepsin, or furin [32, 33]. Alternatively, the viral envelope of SARS-CoV-2 could directly fuse with host cell membranes to enter the cell [34].

With respect to neurons, coronaviruses have been found to be able to reversibly enter the CNS, *via* nerve endings and neuronal active transport between peripheral and central nerves, which are connected by interactions between neurotransmitters and receptors in the postsynaptic membranes [35, 36]. Indeed, neuroinvasive pathways have been documented for HEV67 [37, 38] and OC43-CoV [39] coronaviruses, in which they initially infiltrate peripheral nerve terminals, followed by antero-and retrograde spread *via* CNS synapses [37, 40, 41]. In light of these observations, the most likely peripheral nerve terminal for SARS-CoV-2 neuro-invasion into the CNS is the olfactory nerve, owing to its close proximity to the olfactory epithelium [42]. This is further supported by transmembrane protease, serine 2 (TMPRSS2) and ACE-2 receptors, which are essential for viral binding and entry, being abundantly expressed in olfactory epithelium cells [43]. Therefore, as outlined in a previous study, the S protein of SARS-CoV-2 binds to neuronal ACE-2 receptors and connects to the target cell surface, which is followed by S protein activation/cleavage by the TMPRSS2 serine protease to facilitate viral entry into the neuron [44]. Indeed, another recent study found that SARS-CoV-2 enters the CNS by bridging the neural-mucosal interface, and then penetrating neuroanatomical regions that are receiving inputs from the olfactory tract. This olfactory-based infection pathway is backed by findings of SARS-CoV-2 RNA and distinctive coronavirus-associated substructures within nasal mucus and epithelial cells, as well as a colocalization analysis showing perinuclear S protein positivity within TuJ1^+^, NF200^+^, and OMP^+^ neural cells in the olfactory mucosa, indicating viral infection of olfactory neurons [45, 46]. Furthermore, the olfactory epithelium contains a high concentration of ACE-2 receptors. As a result, SARS-CoV-2 infection and propagation from the nose and cribriform plate of the ethmoid bone to the olfactory epithelium [47], olfactory nerve, and CNS could serve as the underlying basis behind taste and smell abnormalities in COVID-19, though this hypothesized infection pathway has not been fully confirmed [48]. Nevertheless, SARS-CoV-2 infection of the olfactory epithelium serves as the likely cause behind COVID-19 anosmia and could also act as a vector for its CNS entry.

Aside from the olfactory nerve, viral retrograde axonal transport may also occur *via* other cranial nerves, including vagus, glossopharyngeal, and trigeminal neurons [49]. In particular, the vagus nerve, a component of the enteric nervous system associated with the digestive tract, contains ACE-2 and neuropilin-1 (NRP-1) receptors [50], and both antero- and retrograde viral transmission between brainstem neurons and duodenal cells have been reported in a previous study [51]. Consequently, SARS-CoV-2 infection of enterocytes could be followed by their migration to enteric nervous system glial and neuronal cells, and subsequently the CNS, *via* the vagus nerve [42, 52], as the thalamus, and medullary nuclei of the dorsal vagal complex in the brain stem, have been found to be the target of secondary viral dissemination after the initial lung infection [53]. Further research analyzing vagal and human glossopharyngeal neurons at the medulla oblongata level has found that ACE-2 and NRP-1 receptors are abundantly expressed in axons, myelin sheaths, nerve bundles, as well as their supporting cells, which also express TMPRSS2 [54]. With respect to the trigeminal nerve, post-mortem investigations of COVID-19 patients revealed axonal degeneration and cell death there, as well as high SARS-CoV-2 RNA levels in the trigeminal ganglia [45, 55].

Another major infection route is *via* hematogenous (blood) access, which has been documented by Tseng *et al.*, who found that 2 days after SARS-CoV-2 intranasal injection, high viral titers and low-level viremia were detected within the brain [56], as well as another post-mortem analysis identifying SARS-CoV-2 in association with lung capillary damage, therefore allowing pulmonary microcirculation [57]. Furthermore, Zeng *et al.* analyzed blood samples from >100 COVID-19 patients and found that 41% tested positive for SARS-CoV-2 RNA [58]. One of the most common viral entry sites into the CNS is through the blood-brain barrier (BBB) [59], which divides brain parenchyma from plasma. Due to SARS-CoV-2 being able to infect endothelial cells in cerebral blood vessels *via* binding to the ACE-2 receptor, it is therefore able to disrupt the BBB and increase its permeability, in turn causing cerebral edema and intracranial hypertension [60]. This is due to the BBB being mostly comprised of capillary endothelial cells; the tight junctions between them ensure that harmful compounds are unable to enter the brain. Additional cells comprising the BBB include microglia, pericytes, and astrocytes; the “end feet” of astrocytes are a crucial component of the BBB, as they cover large portions of the intracranial arteries [61]. The bypassing of viruses through the BBB has been termed the “Trojan Horse mechanism”, involving viral infection and transcytosis across endothelial cells, as well as leukocyte filtration and recruitment [62]. In the case of SARS-CoV-2, Wang *et al.*, in a cortical organoid study, observed that pericyte-like cells could serve as infection sites, while the basement membrane components synthesized by astrocytes could serve as a hub for viral replication. Therefore, SARS-CoV-2 could cause astrocyte death, pericyte- like cell infection and trigger an inflammatory type I interferon transcription response [63].

### 2.3 Autoimmunity

Persistent neuronal dysfunction has also been linked to COVID-19-linked cytokine storms, triggered by copious amounts of proinflammatory cytokines and chemokines being produced [64, 65], as well as inflammation [23]. In fact, a recent meta-analysis found that heart rate variability (HRV) was inversely correlated to inflammation [66] and could be used as a measure of dysautonomia [67], owing to inflammatory elements being able to traverse the compromised BBB and harm the brain. For instance, individuals with acute respiratory distress syndrome (ARDS) or severe sepsis frequently possess lower HRV, leading to some researchers investigating whether vagus nerve stimulation could serve as a potential therapeutic approach [68]. SARS-CoV-2 has also been found to possibly damage inflammatory cells, such as macrophages, microglia, and astrocytes, as indicated by higher plasma glial fibrillary acid protein (GFAP) and neurofilament light chain (NfL) levels, which are associated with, respectively, greater astrocyte activation/injury and axon damage. These observations therefore illustrate that SARS-COV-2 could inflict neurological harm [69, 70] and that viral-caused inflammation could result in chronic neuronal dysregulation [23]. However, the precise mechanisms by which the ANS and immune system interact to affect HRV are still largely unknown [71].

Cytokine storms result from SARS-CoV-2 superantigens triggering a dysregulated immune system to quickly release a variety of cytokines, in turn increasing their blood circulation concentrations and subsequently leading to various clinical manifestations associated with massive multi-organ destruction and death from exuberant hyperinflammation, at both local and systemic levels [40]. More specifically, NLR family pyrin domain-containing protein 3 (NLRP3) inflammasomes, a key trigger behind cytokine storms, could cause necroptosis, apoptosis, and pyroptosis; in particular, pyroptosis, a type of caspase-dependent inflammatory cell death, could significantly affect CNS disease onset and progression. One significant pyroptosis mechanism involves the N-terminal domain of Gasdermin D [72], which binds membrane lipids, inositol phosphate, and cardiolipin, being separated by activated caspase-1 during NLRP3 inflammasome-mediated pyroptosis [73]. This separated N-terminal domain, in turn, creates Gasdermin holes in the plasma membranes of infected cells, thereby causing pyroptosis. These pyrolyzed cells then produce a multitude of endogenous chemicals that facilitate inflammation and fortify host immune responses. These NLRP1- and NLRP3 inflammasome-mediated neuronal death and pyroptotic processes, previously associated with Alzheimer's disease [74, 75], could thus also be linked to COVID-19-related neuronal injury (Table **1**) [76].

## 3 COVID-19 AND FEMALE FERTILITY

SARS-CoV-2 has also been found by a number of researchers to be linked to impaired female fertility [77, 78]. This may be owed to ACE-2 being expressed by the uterus, ovaries, fallopian tubes, vagina, and placenta, which, along with higher RAS component concentrations, such as Ang1-7 in theca-interstitial cells, renders them vulnerable to SARS-CoV-2 [77]. In fact, both ACE-2 and Ang1-7 are present at every follicular development stage, indicating that they are crucial fertility molecules [77]. Indeed, ACE2 is crucial for ovarian function, as it is responsible for stimulating steroid production [79], follicle formation [80], oocyte growth [81], regulating ovulation [82], and preserving corpus luteum functionality [83]; it is also involved with endometrial tissue changes and embryo development [77]. As a result, SARS-CoV-2 may impair female fertility *via* its destruction of endometrial epithelial, ovarian, and granulosa cells [78], as well as producing proteins that could trigger NLRP3 inflammasome assembly [84, 85]. This inflammasome, as a crucial component of the innate immune system, serves as one of the initial lines of defense against viral infections. Furthermore, NLRP3 activates Caspase-1 and subsequently increases interleukin (IL)-1β and -18 production [86, 87]. Given the observation that NLRP3 and proinflammatory cytokines are more prevalent within the endometrium of women who have undergone repeated miscarriages [88], it is therefore plausible that SARS-CoV-2-related inflammation could negatively affect female fertility. Indeed, an observational, single-center investigation of 78 reproductive-aged females found that COVID-19-infected women showed signs of ovarian damage, including decreased ovarian reserve and reproductive endocrine dysfunction [89].

During pregnancy, RAS components have been found in the plasma as early as 6 weeks of gestation [90], particularly in the fetal capillaries of main and secondary villi, cytotrophoblasts, syncytiotrophoblasts, as well as maternal decidua and spiral arteries. Additionally, ACE-2 is expressed by umbilical cord vascular endothelium and smooth muscle [90]. However, the role of RAS in the placenta is still unclear. It has been proposed that alterations in placental RAS expression may be associated with faulty placentation, which could cause intrauterine growth limitation or pre-eclampsia [91]. Furthermore, a number of investigations have documented that SARS-CoV-2 infection could heighten oxidative stress [92, 93], which could negatively affect oocyte and embryo quality and correspondingly, female fertility [94-96]. This has been supported by Wang *et al.*, finding that SARS-CoV-2 infection was associated with notably reduced blastocyst formation rates, despite comparable oocyte counts, rates of fertilization, implantation, abortion, and clinical pregnancies to that of healthy mothers [97].

Another key receptor facilitating SARS-CoV-2 entry into host cells is basigin (BSG) [98, 99], which has been implicated in corpus luteum formation, follicle development, and embryo implantation [100]. BSG is expressed by ovary stroma, cumulus, and granulosa cells, and both BSG mRNA and protein have been detected within the corpora lutea and granulosa cells of follicles at every developmental stage [101]. BSG has also been found to facilitate HIV-1, malaria, and *Neisseria meningitides* infections [102]. Moreover, both embryonic and uterine BSG expression has been associated with successful implantation, and disruption/inhibition may result in deficiencies in embryo implantation [103, 104]. Furthermore, COVID-19-caused immune system impairment may alter hypothalamic-pituitary-gonadal axis functioning to alter progesterone and androgen levels [105, 106], which could lead to a positive feedback loop, as sex hormones are powerful immune modulators. As a result, sex hormone level changes could further exacerbate inflammatory and immunological responses to COVID-19 [107].

COVID-19 could also affect the coagulation cascade of pregnant individuals, as normal pregnancy could induce hypercoagulability, which could be exacerbated by the disease to pathological levels. During normal pregnancy, to prevent postpartum hemorrhage, there is increased synthesis of thrombin and prothrombotic factors, including fibrinogen, von Willebrand factor, factors VII, VIII, X, and XII, as well as altered fibrinolysis. On the other hand, the anticoagulant protein S, as well as ACE levels [108], decreases [109], while several RAS components are overexpressed during pregnancy, including Ang1-7, which acts as a vasodilator [110]. All of these pregnancy-associated alterations in RAS and coagulation factors, though, have to be finely-balanced in order to avoid any pathological coagulation. These processes could be disrupted by COVID-19, owing to alterations in RAS and inflammatory responses that could further exacerbate hypercoagulation, possibly leading to thrombi formation and embolism.

**Fig. (1):**
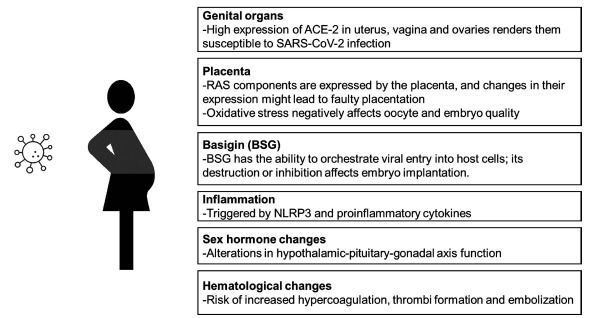
SARS-CoV-2 negatively effects female fertility *via* multiple mechanisms, such as destroying uterine, vaginal, and ovarian cells owing to the virus binding to angiotensin converting enzyme (ACE)-2 receptors, altering renin-angiotensin-aldosterone system (RAS) component expression to result in faulty placentation and oxidative stress to negatively affect oocyte/embryo quality, inhibiting BSG to prevent embryonic implantation, activating the NLRP3 inflammasome to cause inflammation, as well as increased risk for hypercoagulation and alterations in hypothalamic-pituitary-gonadal axis function.

Taken together, these findings thus indicate that SARS-CoV-2 infection negatively affects female fertility through multiple pathways, such as faulty placentation *via* altering RAS component expression, triggering oxidative stress that degrades oocyte/embryo quality, destruction of follicular and endometrial cells, preventing embryo implantation by inhibiting BSG, and activating the NLRP3 inflammasome (Fig. **1**).

## 4 TREATMENTS

### 4.1 Pharmacological Treatments

General pharmacological treatments for treating COVID-19 include midocaine, methylphenidate, or octreotide to induce vasoconstriction, hydrocortisone fluoride, erythropoietin, and despressin to raise blood volume, beta-blockers, metoprolol, or ivabradine to treat tachycardia [111], as well as pyristoxamine to stimulate synaptic transmission [17, 112, 113]. Additionally, pyristine, midodrine, or hydroxydopamine are prescribed for hypotensive individuals, while patients with low blood volume typically receive intravenous saline and intravascular dilation, though hydrocortisone and despressin are better suited for those with severe refractory symptoms. Sympathetic medications, such as clonidine and methyldopa, may be administered to individuals with hyperadrenergic conditions, including tachycardia [114]. Despite the variety of treatment options, further research is required to clarify the underlying processes of dysautonomia, so that targeted treatment approaches can be developed [11].

If a SARS-CoV-2-infected patient exhibits CNS symptoms, active antiviral therapy, combined with symptomatic supportive care, should be performed to stabilize the internal environment and preserve vital signs. Several antiviral drugs have been identified as potentially significant in the SARS-CoV-2 treatment. Among these, Chloroquine (CQ) and Hydroxychloroquine (HCQ) have demonstrated enormous promise in treating COVID-19-related pneumonia in recent clinical trials [26].

There, it is important to focus on lowering cerebral hypertension using dehydrating medications, such as furosemide and mannitol. Furthermore, these patients are also at risk for abrupt respiratory failure [40], necessitating the reduction of respiratory symptoms, as well as avoiding acute respiratory failure. Therefore, improving COVID-19 prognoses among these patients likely depends on preventing and treating CNS infections. Numerous medications have also been developed to lessen the likelihood of COVID-19-associated neurological damage, such as the NLRP3 inhibitor MCC950, an FDA-approved oral medication to treat inflammation [115]. Other therapeutic strategies to prevent the neurological side effects include inhibitors of the main protease of SARS-CoV-2 (M^pro^) [116], as well as those inhibiting RIPK3 or RIPK1; indeed, a number of RIPK1 inhibitors are now undergoing clinical trials [117].

### 4.2 Nonpharmacological Measures

**Fig. (2):**
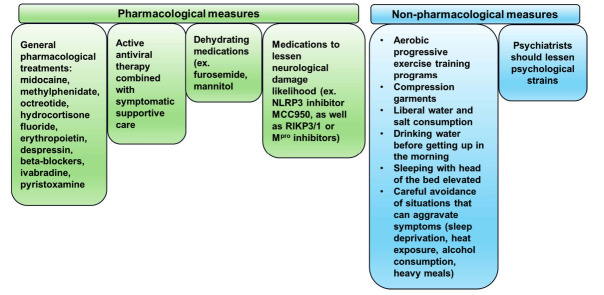
Pharmacological and non-pharmacological measures for treating COVID-19. Pharmacological measures include general treatments, ranging from midocaine, methylphenidate, octreotide (vasoconstriction), hydrocortisone fluoride, erythropoietin, despressin (raise blood volume), beta-blockers, ivabradine (tachycardia), and pyristoxamine (stimulate synaptic transmission). They also include active antiviral therapy combined with symptomatic supportive care, dehydrating medications to lower cerebral hypertension, as well as medications to lessen the likelihood of COVID-19-associated neurological damage, such as the NLRP3 inhibitor MCC950, an FDA-approved oral medication to treat inflammation. Non-pharmacological measures include aerobic progressive exercise, compression garments, liberal water and salt consumption, careful avoidance of situations that can aggravate symptoms, and psychological therapies.

Non-pharmacological measures should also be applied for treating COVID-19, including physical reconditioning with aerobic progressive exercise training programs [15, 118], compression garments [119], liberal water and salt consumption, drinking water before getting up in the morning, sleeping with the head of the bed elevated [120], and careful avoidance of situations that can aggravate symptoms (sleep deprivation, heat exposure, alcohol consumption, heavy meals) [16]. Additionally, in order to prevent COVID-19 symptoms from worsening, psychological therapies should be administered to lessen the mental strain of the disease (Fig. **2**) [121].

### 4.3 Sex-based Differences in Treatment

There is a need to examine the impact of sex and gender variations on COVID-19, as well as devise specialized medical regimens, as studies have revealed that males and females experience the disease differently [122]. Some of those male/female differences are connected to sex-related biological parameters (sex hormones and chromosomes), while others are associated with gender [123-126]. More specifically, XX chromosomes, as well as estrogen, have been found to lessen COVID-19 severity among females, though this protective effect is lost with age [123-126]. Therefore, reasonable selection of the timing, administration mode and formulation of estrogen replacement therapy may have more beneficial effects on the cardiovascular system and, in turn, mitigate COVID-19-associated adverse cardiovascular outcomes [126].

### 4.4 COVID-19 Vaccine

One of the most important ways to stop the spread of SARS-CoV-2 and eventually stop the pandemic is through vaccination. Inactivated, live attenuated, viral vector, protein subunit, RNA, DNA, and virus-like particle (VLP) vaccines are the most common types of SARS-CoV-2 vaccines now in use. Strong cellular and humoral immunity may be triggered by the COVID-19 vaccine-induced memory cell responses, which can also produce Th1 and persistent germinal center responses. Antigen-specific memory T cells and B cells are often produced during this phase, which is important for long-term immunity [127]. Following COVID-19 vaccination, one may experience the production of T-cell immunity (like the Th1 cell response), B-cell immunity (like the germinal center response), and other immunological responses [128, 129]. Activating CD8^+^ T cells and secreting IFN-γ are two ways that differentiated Th cells might improve the body's immunological response [130]. Activated B cells develop and multiply in lymphatic follicles to create germinal centers, which in turn generate plasma cells, and memory B cells secrete high-affinity antibodies with the help of Th cells. Furthermore, COVID-19 vaccinations can generate memory T and B cells [131]. The humoral immune response, cellular immune response, and memory cells work together to build the antiviral immunological barrier in the host body.

Studies have shown that adverse events such as miscarriage or fetal abnormalities may occur after vaccination against COVID-19 [132, 133]. Shimabukuro *et al.* [133] used the VERS and V-safe monitoring systems to assess the impact of the COVID-19 vaccine on expectant mothers and fetuses. According to the findings, pregnant women experienced more unfavorable effects than non-pregnant women. 13.9% of the pregnant women had pregnancy loss following the mRNA immunization, 86.1% experienced a normal pregnancy, and 9.4% delivered their baby early. Although there is a small chance of both pregnancy loss and early delivery, immunization has considerably more advantages than disadvantages. Furthermore, research has demonstrated that pregnant women who receive vaccinations have a protective impact on themselves and create antibodies that may be transferred to the fetus through the umbilical cord or breast milk, so offering immunological protection [134, 135]. Additionally, a multicenter trial carried out in Israel showed that the mother might develop IgG antibodies following immunization with the BNT162b2 vaccine. Newborns are capable of detecting antibody responses, and these antibodies can cross the placental barrier [135]. These two investigations demonstrated that COVID-19 immunization might successfully protect the fetus by transferring antibodies from the mother to the kid through efficient mother-to-child transmission.

## CONCLUSION

Hypovolemia, brain stem involvement, and autoimmunity have been suggested as 3 potential factors that may lead to autonomic dysfunction caused by SARS-CoV-2. Increased SNS outflow from insufficient blood volume may lead to POTS and may also disrupt the central autonomic neural network, resulting in increased sympathetic outflow in response to central autonomic activation. SARS-CoV-2 causes brain stem dysfunction through direct viral invasion, neuroinflammation, and vascular activation. In addition, the cytokine storm associated with COVID-19 also causes persistent neuronal dysfunction. This dysfunction, however, can be treated with drug and non-drug treatment programs, estrogen replacement therapy and vaccination. Further investigation is required to clarify the underlying causes of autonomic dysfunction, and, consequently, guide possible alternative therapeutic approaches. For COVID-19 and female fertility, while the proportion of adverse events following immunization that affects pregnant women is higher than that of non-pregnant women, it is still quite small. The COVID-19 vaccination can, within an optimum range, protect both mothers and babies at the same time, lowering the likelihood of fetal infection with SARS-CoV-2 after birth. And these new treatments will undoubtedly be a powerful weapon in the fight against COVID-19 and help end the pandemic.

## Figures and Tables

**Table 1:** The 3 autonomic dysfunction mechanisms associated with long-COVID.

**Autonomic Dysfunction Mechanism**	**Specific Content**	**References**
Hypovolemia	• Patients with COVID-19 symptoms frequently express hypovolemia due to increased cardiac sympathetic noradrenergic system (SNS) outflow. • Hypovolemia could cause hyperadrenergic postural orthostatic tachycardia syndrome (POTS) due to disruptions in central autonomic networks.	[13-21]
Brain stem involvement	• SARS-CoV-2 may enter the central nervous system (CNS) *via* the olfactory nerve after entering the olfactory epithelium. The olfactory epithelium is able to serve as a vector for the virus to invade the CNS owing to it expressing angiotensin converting enzyme (ACE)-2 receptors. • Retrograde axonal transport of the virus may be possible through other cranial nerves, including vagus, glossopharyngeal, and trigeminal nerves. • SARS-CoV-2 also binds to ACE-2 protein expressed in endothelial cells in the blood-brain barrier, disrupting the cells there, which leads to cerebral edema and intra-cranial hypertension.	[22-63]
Immune-mediated mechanisms	• Cytokine storms and inflammation, brought on by COVID-19 infection, may result in persistent neuronal dysfunction.	[23, 64-76]

## LIST OF ABBREVIATIONS

ANS: Autonomic Nervous System
COVID-19: Coronavirus Disease 2019
SARS-CoV-2: Severe Acute Respiratory Syndrome Coronavirus 2
CNS: Central Nervous System
SNS: Sympathetic Noradrenergic System
CSF: Cerebrospinal Fluid
ARDS: Acute Respiratory Distress Syndrome
GFAP: Glial Fibrillary Acid Protein
